# Altered expression of members of the IGF-axis in hepatoblastomas

**DOI:** 10.1054/bjoc.1999.1179

**Published:** 2000-04-03

**Authors:** S G Gray, T Eriksson, C Ekström, S Holm, D von Schweinitz, P Kogner, B Sandstedt, T Pietsch, T J Ekstöm

**Affiliations:** Laboratory for Molecular Development and Tumour Biology, Experimental Alcohol and Drug Addiction Research Section, Dept. of Clinical Neuroscience, Karolinska Institute, CMM L8 01, Stockholm, S-171 76, Sweden; Department of Pediatric Surgery, University of Basel Childrens Hospital, PO Box CH-4005, Basel, Switzerland; Childhood Cancer Research Unit, Department of Woman and Child Health, Karolinska Hospital, Stockholm, Sweden; Pathology Department, Pediatric Division, Karolinska Hospital, Stockholm, Sweden; Department of Neuropathology, University of Bonn Medical Center, Bonn, D-53105, Germany

**Keywords:** hepatoblastoma, insulin-like growth factors, binding proteins

## Abstract

Previous reports have demonstrated that expression of insulin-like growth factor 2 (*IGF2*) is altered in hepatoblastoma. Using RNAase protection analysis (RPA), we examined the gene expression for *IGF1, IGF2, IGF1R, M6P/IGF2R, IGFBP-1* and *IGFBP-2* in a series of hepatoblastomas with corresponding normal liver from the same individuals. The results show that the expression of the IGF-axis members included in the present study are altered between tumour and normal, and indicate that the IGF-axis may be involved in hepatoblastoma development. © 2000 Cancer Research Campaign


*British Journal of Cancer* (2000) **82**(9), 1561–1567
© 2000 Cancer Research Campaign
DOI: 10.1054/ bjoc.1999.1179, available online at http://www.idealibrary.com on IDEAL®

### Altered expression of members of the IGF-axis in hepatoblastomas

**SG Gray[1], T Eriksson[1], C Ekström[1], S Holm[1], D von Schweinitz[2], P Kogner[3], B Sandstedt[4], T Pietsch[5] and TJ Ekström[1]**

[1]Laboratory for Molecular Development and Tumour Biology, Experimental Alcohol and Drug Addiction Research Section, Dept. of Clinical Neuroscience, Karolinska Institute, CMM, L8 01, S-171 76 Stockholm, Sweden; [2]Department of Pediatric Surgery, University of Basel Childrens Hospital, PO Box CH-4005, Basel, Switzerland; [3]Childhood Cancer Research Unit, Department of Woman and Child Health, Karolinska Hospital, Stockholm, Sweden; [4]Pathology Department, Pediatric Division, Karolinska Hospital, Stockholm, Sweden; [5]Department of Neuropathology, University of Bonn Medical Center, D-53105 Bonn, Germany

**Summary** Previous reports have demonstrated that expression of insulin-like growth factor 2 (*IGF2*) is altered in hepatoblastoma. Using RNAase protection analysis (RPA), we examined the gene expression for *IGF1, IGF2, IGF1R, M6P/IGF2R, IGFBP-1* and *IGFBP-2* in a series of hepatoblastomas with corresponding normal liver from the same individuals. The results show that the expression of the IGF-axis members included in the present study are altered between tumour and normal, and indicate that the IGF-axis may be involved in hepatoblastoma development. © 2000 Cancer Research Campaign

**Keywords:** hepatoblastoma; insulin-like growth factors; binding proteins

Hepatoblastoma is a rare malignant childhood tumour of the liver and accounts for approximately 1–2% of all malignant tumours in children. The tumour is believed to be embryonic in origin, and accounts for more than 25% of all paediatric hepatic tumours and for nearly 50% of malignant liver neoplasms in this age group (Sainati et al, 1998). Although characterized by a wide spectrum of subtypes, the majority of hepatoblastomas are composed principally of epithelial cells that resemble fetal and embryonal hepatocytes which are often admixed with mesenchymal cells (von Schweinitz et al, 1994). The prognosis for affected children has improved drastically in the last few years but even so, approximately 25% of all affected children do not survive the disease.

Loss of heterozygosity (LOH) at 11p has been extensively studied for the chromosomal region 11p15.5 in hepatoblastomas. This region contains the insulin like growth factor (*IGF2*) and *H19* (a putative tumour supressor) genes, both of which have been shown to be important in tumorigenesis (for reviews see De Souza et al, 1997; Looijenga et al, 1997). Both genes are subject to a phenomenom known as genomic imprinting, a situation in which expression of a gene is dependent upon the parent of origin (for reviews see Franklin et al, 1996; Constancia et al, 1998). Loss of imprinting (LOI) and LOH have been reported for *IGF2* and *H19* in hepatoblastoma (Albrecht et al, 1994; Montagna et al, 1994; Li et al, 1995; Rainier et al, 1995). The expression of these genes have also been shown to be altered in hepatoblastoma. We and others have observed that *H19* was down-regulated in hepatoblastomas (Albrecht et al, 1994; Montagna et al, 1994; Li et al, 1995) whilst some studies have seen no alteration in its expression (Rainier et al, 1995; Yun et al, 1998).

IGF-II plays a key role in mammalian growth and fetal cell division (Odell and Day, 1998), and its expression is frequently altered in cancers and overgrowth disorders (Morison and Reeve, 1998).

*IGF2* contains four promoters (P1–P4) which are utilized in a developmental and tissue specific fashion. In hepatoblastomas, expression from promoters 1 and 4 were shown to have decreased, whilst that of promoters P2 and especially P3 were up-regulated (Li et al, 1995). Up-regulation of *IGF2* was originally observed to be occurring in poorly or moderately differentiated hepatoblastoma cells and in those tumours associated with epithelial differentiation (Akmal et al, 1995). A recent study showed that the expression of *IGF2* (at least for promoters 1 and 3) occurs in hepatocytes surrounding the central vein. No expression of *IGF2* was observed in haematopoietic, bilary duct or vascular endothelial cells (Yun et al, 1998).

A series of proteins which affect the insulin-like growth factors is the insulin-like growth factor binding protein family. This family consists of two subgroups, with six insulin-like growth factor binding proteins (IGFBPs 1–6), and nine insulin-like growth factor binding protein related proteins (IGFBP-rPs 1–9), whose common property is their ability to bind insulin-like growth factors 1 and 2 (IGF-1 and IGF-2) and modulate many aspects of the IGF-axis (Wetterau et al, 1999). Overexpression of IGFBP-2 has been previously observed in hepatoblastoma correlating with the degree of tumour cell differentiation (Akmal et al, 1995).

In this study, we examined the expression of several members of the IGF-axis in a series of well characterized hepatoblastomas with corresponding normal liver tissue taken from the same individual for most of the samples. In most of these cases, the results show that the primary difference between normal liver tissue and hepatoblastoma tissue is a reduction in IGF-binding protein mRNA levels.

*Received 9 September 1999*
*Revised 29 November 1999*
*Accepted 9 December 1999*

*Correspondence to:* TJ Ekström

**Table 1** Samples used in this study

| Case | Age (month)/ sex | Pre-operative chemotherapy | Histology | Other features | 1p LOH | 11p LOH | Outcome |
|---|---|---|---|---|---|---|---|
| Matched pairs | | | | | | | |
| HB 1 | 6♂ | No | Epithelial | | No | Yes | NED |
| HB 2 | 19♂ | Yes | Epithelial | Liver tissue with bile stasis and multi-focal regions of hepatoblastoma | No | Yes | DOD |
| HB 3 | 19♂ | Yes | Epithelial | Connective tissue present. Proliferating bile ducts | No | No | NED |
| HB 4 | 22♂ | Yes | Mixed Epithelial/mesenchymal | Liver tissue with fibrosis present | No | No | NED |
| HB 5 | 54♂ | Yes | Epithelial | Connective tissue present | Yes | No | NED |
| HB 6 | 2♂ | No | Fetal | Vacuolized cytoplasm. Extra-medullary haematopoesis. Metaplastic osteoids | ? | ? | NED |
| HB 7 | 12♀ | Yes | Fetal | Hepatocytes show trabecular and acinary cell arrangment. Apoptotic cells with lymphocyte infiltration, macrophages, proliferating capillaries and foci of haematopoesis | ? | ? | NED |
| HB 8 | 36♂ | Yes | | Hepatoblastoma. No further information | ? | ? | NED |
| Unmatched tumours | | | | | | | |
| HB 9 | 11♀ | No | Fetal | Some connective tissue septa | No | No | NED |
| HB 10 | 13♂ | No | Epithelial | Well differentiated hepatoblastoma | No | No | NED |
| HB 11 | 8♀ | No | Mixed Epithelial/mesenchymal | Some streaks of bone tissue | No | No | NED |
| Fetal Liver | | | | | | | |
| 7 week | N/A | N/A | Normal | | N/A | N/A | N/A |
| 13 week | N/A | N/A | Normal | | N/A | N/A | N/A |
| 14 week | N/A | N/A | Normal | | N/A | N/A | N/A |

Symbols used: ♂ – male; ♀ – female; LOH – loss of heterozygosity; NED – no evidence of disease; DOD – dead of disease; ? – Unknown; N/A – not applicable.

### MATERIALS AND METHODS

### Samples

All tumours with the exception of the HB6, HB7 and HB8, were freeze-sectioned into 1 mm portions interrupted by 5 μm sections. The 1 mm sections numbered consecutively were used for RNA isolation, while the interrupted thin sections were prepared for histopathological examination. In this way good tissue profiles were obtained. The histopathological examinations made at the Perinatal Pathology Section at the Karolinska Hospital gave results as shown in Table 1.

Human fetal livers (7, 13 and 14 weeks old), were obtained from therapeutic terminations, with the permission of the local ethical committee. Due to the nature of such procedures limited amounts of such tissues were obtained. For this reason, we were unable to include any RNA from these samples in the analysis of *M6P*/*IGF2R* and *IGFBP-1*.

### Nucleic acid isolation

Total RNA was prepared as described previously (Chomczynski and Sacchi, 1987).

### Preparation of probe and RNase protection analysis (RPA)

RNA probes were prepared from the above templates using T3 and T7 RNA polymerases (Life Technologies) according to the protocol provided in the RPA II Kit (Ambion). When incorporating radioactivity into the probe, radioactive $^{32}$P-UTP with a specific activity of 800 Ci mmol$^{-1}$ was used. Cold UTP was added such that final probe activity was 400 Ci mmol$^{-1}$ for all probes except 80 Ci mmol$^{-1}$ for the *GAPDH* probe.

The probes used in this study are as follows:

- Probes used to examine *H19*, total *IGF2*, *IGF2* promoter P1, *IGF2* promoter P4 and *IGF1* were generated as described previously (Ohlsson et al, 1994; Ekström et al, 1995; Li et al, 1995, 1998*a*, 1998*b*; Olivecrona et al, 1999).
- To examine *IGF2* promoter P2-specific transcripts, a *Pst* I–*Sma*I fragment covering the 5′-end of *IGF2* exon 4 was cloned into pBluescript SK II$^-$ vector (Stratagene, La Jolla, CA, USA). When this plasmid was linearized with *Eco*RI, T3 RNA polymerase was used to transcribe a probe of 311 bases. When used in RNAase protection analysis, 220 bp hybridized to *IGF2* P2-specific mRNA transcripts and was protected from digestion.
- For *IGF2* promoter P3-specific mRNA transcripts a blunted *Sma*I–*Bgl*I specific to the 3′-end of *IGF2* exon 5 was cloned into the *Eco*R V site of pBluescript SK II$^-$ vector (Stratagene). After linearization of the resulting plasmid with *Eco*RI, T7 RNA polymerase could be used to transcribe a 298 bp RNA probe. When used in a hybridization reaction, 111 bp of this probe hybridizes specifically to *IGF2* P3 mRNA transcripts.
- The *M6P*/*IGF2R* probe used in this series of experiments was generated from plasmid p146 as previously described, which allows the detection of the ACAA+/– polymorphism (Smrzka et al, 1995). When linearized with *Hin*dIII an RNA probe of 269 bp could be generated with T3 RNA polymerase of which either 147 bases (ACAA+) or 125 bases (ACAA–) will hybridize to *M6P*/*IGF2R*-specific transcripts.

 *© 2000 Cancer Research Campaign*

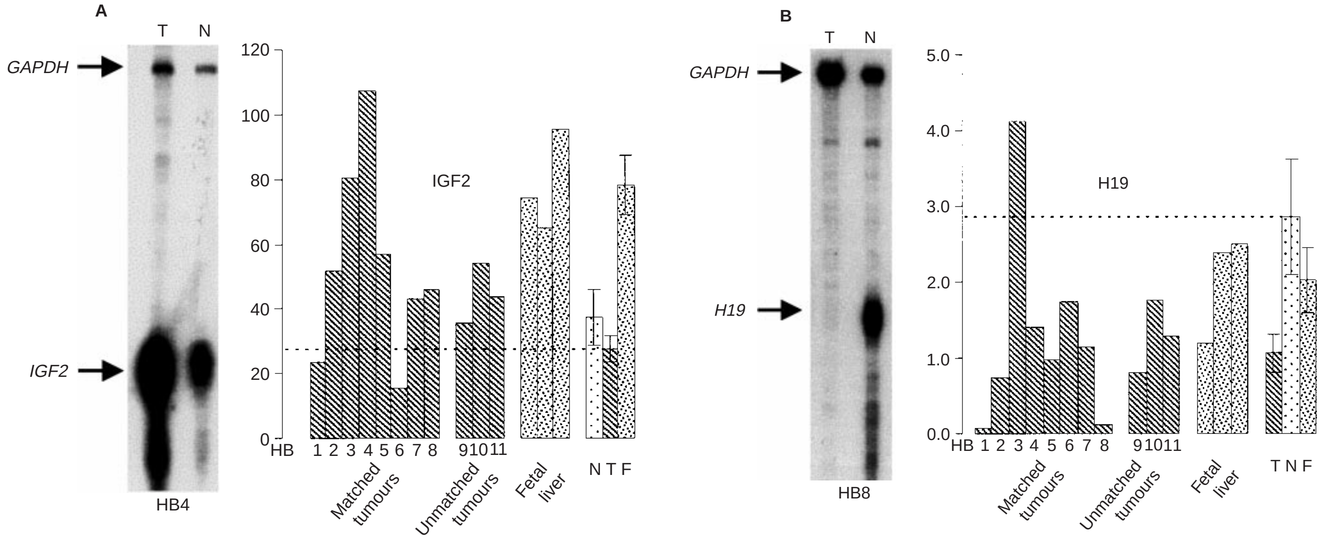

**Figure 1** RNase protection analysis of *IGF2* and *H19* expression. (**A**) Analysis of total *IGF2* transcripts in hepatoblastomas. A single representative RNase protection analysis for one of the matched tumours is shown. *GAPDH* expression is used as the internal control for quantification purposes. In all of the following figures the *Y*-axis units represent the values for each gene divided by the value obtained for the housekeeping gene *GAPDH* (in this case: *IGF2/GAPDH*) as determined by phosphorimager analysis and following the adjustments as described in Materials and Methods. The mean ± standard error of the mean was also calculated for the tumours (T), normals (N) and fetal tissues (F), and graphed along with the individual samples. Matched tumours are those samples for which normal liver was taken from the same individual at time of surgery. Unmatched tumours are those samples for which normal liver tissue was unavailable. Fetal livers were included to compare against normal liver and tumour expression. (**B**) Analysis of *H19* expression in hepatoblastomas. A representative RNase protection analysis showing total *H19* transcripts in one of the matched tumours is shown. Following quantification with the internal control (*GAPDH*), *H19* expression for the matched samples was calculated and graphed as described above

- The *IGF1R* probe used in this series of experiments was a gift from Dr Gunnar Norstedt. After digestion of the plasmid with *Pvu*II, T3 RNA polymerase was used to generate a probe with a size of 411 bases. When used in the protection assay 184 bases of these probe transcripts could hybridize specifically to *IGF1R* mRNA.
- The *GAPDH* clone (pTRI-GAPDH-Human) used in these experiments was purchased from Ambion. When hybridized to mRNA this probe protects 315 bases from digestion. RNAase protection was carried out according to the protocol given with the RPA II Kit (Ambion).

### Analysis of expression

Quantification of the results was obtained using phosphor imager analysis (BAS-1000, Fuji Photo Film Co., Ltd) with *GAPDH* mRNA levels utilized as the internal control in each case. In each case the values for the gene under scrutiny were normalized to the internal control. The average value for all the normal samples was obtained and set as the arbitrary value for normal liver. The value obtained for the tumours was therefore adjusted by multiplying the obtained value by the ratio of the arbitrary normal value to the matched normal liver.

*Adjusted tumour value = Tumour value × (average value for all the normals ÷ matched normal value)*

Example

| Sample values | Original value | Average N ($N_{avg}$) | Factor | Adjusted sample values |
|---|---|---|---|---|
| $T_1$ | 2 | 6 | | 4 |
| $N_1$ | 3 | | 2 | 6 |

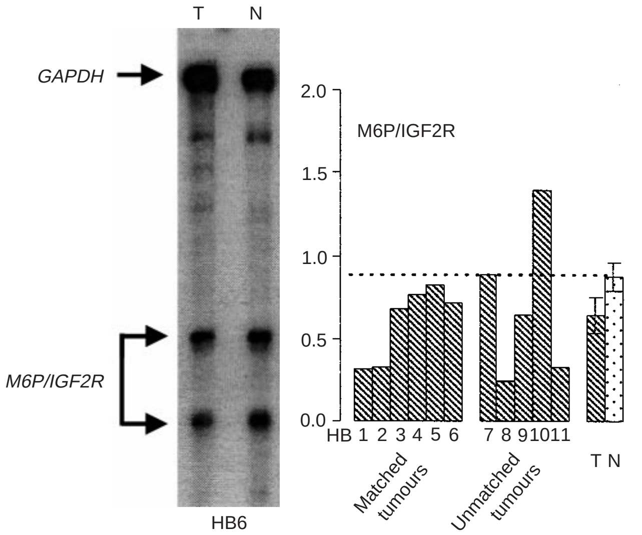

**Figure 2** RNase protection analysis of *M6P/IGF2R* expression. Using RNase protection analysis *M6P/IGF2R* transcripts were quantified and graphed as described in Figure 1. However, due to a lack of available RNA at the time of analysis the samples HB7 and HB8 were included in the unmatched tumours, and no fetal tissues were examined. A representative RNase protection analysis for one of the matched tumours is shown

(eg: average normal ($N_{avg}$) = 6. A tumour ($T_1$) has a value of 2 and its matched normal ($N_1$) has a value of 3. The factor ($N_{avg} \div N_1$) required to bring $N_1$ up to $N_{avg}$ is 2. Both $N_1$ and $T_1$ are therefore multiplied by two to give the final values as indicated $N_1$ = 6 and $T_1$ = 4.

© 2000 Cancer Research Campaign

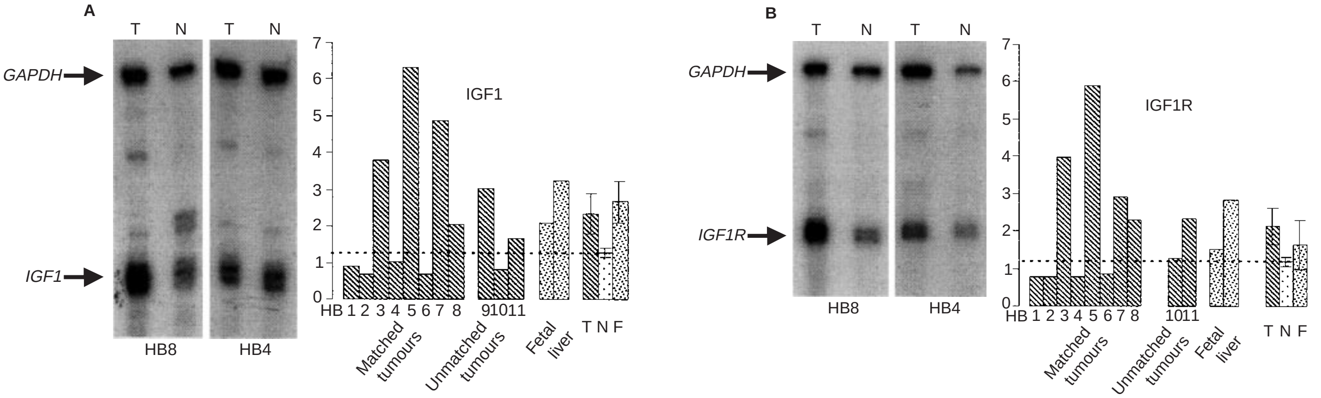

**Figure 3** RNase protection analysis of *IGF1* and *IGF1R* expression. (**A**) Quantification of *IGF1* transcripts in hepatoblastomas. A representative RNase protection analysis is shown showing *IGF1* and *GAPDH* protected fragments for two of the matched tumours. Following quantification, the results were graphed as described in Figure 1. (**B**) Quantitative analysis of *IGFIR* expression. Representative results of the RNase protection analysis for two of the matched tumours are shown. Following quantification with the internal control (*GAPDH*), *IGFIR* expression for the matched samples was calculated and graphed as described in Figure 1

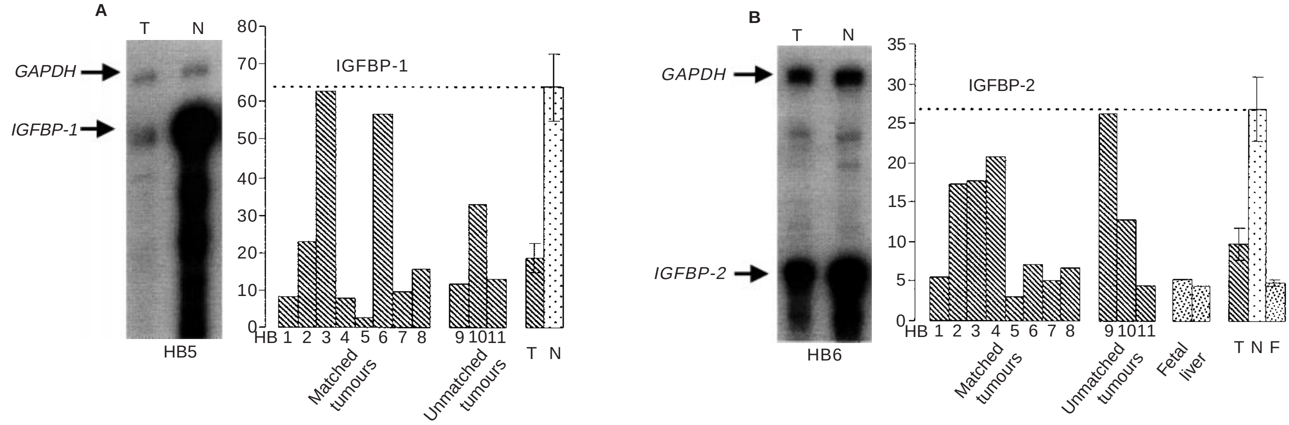

**Figure 4** RNase protection analysis of *IGFBP-1* and *IGFBP-2* expression. (**A**) Quantification of *IGFBP-1* expression. A representative RNase protection analysis showing *IGFBP-1* and *GAPDH* transcripts in one of the matched hepatoblastoma samples is shown. The results were analysed and graphed as detailed in Figure 1. Due to a lack of available RNA no fetal liver was examined for *IGFBP-1* expression. (**B**) Quantitative analysis of *IGFBP-2* expression. A representative result of the RNase protection analysis is shown for one of the hepatoblastoma samples. Following quantification with the internal control (*GAPDH*) by phosphorimager analysis, *IGFBP-2* expression for the matched samples was calculated and graphed as described in Figure 1

The mean ± standard error of the mean was also calculated for the tumours, normals and fetal tissues and graphed along with the individual samples.

### RESULTS

### Expression of *IGF2* and *H19*

We examined the total transcriptional activity, as well as the relative activity from the different *IGF2* promoters, and the expression levels of *H19* in a series of matched hepatoblastomas and the corresponding normal liver tissue from patients between the ages of 2 and 54 months (Table 1). Included in the analysis were a series of hepatoblastomas with no counterpart normal tissues and several fetal liver samples. In accordance with previously published results expression of *IGF2* was increased in most of the tumour samples when compared against corresponding normal tissue, with two exceptions, HB1 and HB6 (Figure 1A) (Li et al, 1995). The human *IGF2* gene is transcribed from four promoters (P1–P4). When *IGF2* promoter usage was examined the pattern of expression which emerged was as follows. The major transcript produced by the hepatoblastomas was from promoter P3, with increased expression from promoter P2, decreased expression from promoter P4, and no expression from promoter P1 (data not shown). These results are in concordance with our previously publsihed results (Li et al, 1995, 1998*B*).

When *H19* expression was examined, similar results to those previously presented were observed (Li et al, 1995). In general, for the hepatoblastomas, *H19* expression was decreased (Figure 1B). The one exception showing increased *H19* expression in this series of experiments is the sample HB3.

### Expression of the *M6P/IGF2R* in normal versus tumour tissue

As *IGF2* was increased in hepatoblastomas, we set out to examine the expression of the mannose-6-phosphate/insulin-like growth factor II receptor (*M6P*/*IGF2R*) in these tissues. One of the roles of this receptor is to bind IGF-II. whereupon it is internalized

 © 2000 Cancer Research Campaign

**Table 2** Results of gene expression analysis in the hepatoblastomas with respect to clinical outcome and histology

| Case | Outcome | Histology | *IGF2* | *H19* | *M6P/IGF2R* | *IGF1* | *IGF1R* | *IGFBP-1* | *IGFBP-2* |
|---|---|---|---|---|---|---|---|---|---|
| HB1 | NED | Epithelial | N | ↓ | ↓ | ↓ | ↓ | ↓ | ↓ |
| HB2 | DOD | Epithelial | ↑ | ↓ | ↓ | ↓ | ↓ | ↓ | ↓ |
| HB3 | NED | Epithelial | ↑ | ↑ | ↓ | ↑ | ↑ | N | ↓ |
| HB4 | NED | Mixed Epithelial/Mesenchymal | ↑ | ↓ | ↓ | ↓ | ↓ | ↓ | ↓ |
| HB5 | NED | Epithelial | ↑ | ↓ | N | ↑ | ↑ | ↓ | ↓ |
| HB6 | NED | Fetal | ↓ | ↓ | ↓ | ↓ | ↓ | N | ↓ |
| HB7 | NED | Fetal | N | ↓ | N | ↑ | ↑ | ↓ | ↓ |
| HB8 | NED | N/A | N | ↓ | ↓ | ↑ | ↑ | ↓ | ↓ |
| HB9 | NED | Fetal | N | ↓ | ↓ | ↑ | N/D | ↓ | N |
| HB10 | NED | Epithelial | ↑ | ↓ | ↑ | ↓ | N | ↓ | ↓ |
| HB11 | NED | Mixed Epithelial/Mesenchymal | N | ↓ | ↓ | ↑ | ↑ | ↓ | ↓ |

Symbols used: NED – no evidence of disease; DOD – dead of disease; N/D – not determined; N – normal expression; ↑ – increased expression; ↓ – decreased expression.

and subsequently degraded by lysosomes (De Souza et al, 1997). The results of this analysis show that for some cases (notably HBs 1, 2, 8 and 11) expression of the receptor is decreased (Figure 2). The degree of expression, however, varies between samples. Several of the tumours show levels of expression which are close to the arbitrary normal value. Also, one sample HB10, shows an increased expression of this gene. Due to a lack of available RNA at the time of analysis the samples HB7 and HB8 were included in the unmatched tumours (Figure 2).

### *IGF1* and *IGF1R* expression in hepatoblastomas

The expression profiles of the genes for *IGF1* and the *IGF1R* were then examined in our samples to see if there were any differences between tumour versus normal tissues. The results are shown in Figure 3. *IGF1* expression showed a varied expression profile.

When the matched tumours are compared against the arbitrary normal, two groups emerge, those that show increased *IGF1* expression (samples HB3, HB5, HB7 and HB8), and those that show decreased *IGF1* expression (HB1, HB2 and HB6) (Figure 3A). The same result was observable if the individual RPA values for each matched tissue set was compared without adjustment (data not shown).

When *IGF1R* expression was examined a similar expression profile emerged (Figure 3B). Those samples which showed increased *IGF1* expression also showed increased *IGF1R* expression and those showing decreased *IGF1* expression correlated with decreased *IGF1R* expression (Figure 3 A, B).

### Expression of *IGFBP-1* and *IGFBP-2*

Previously it was shown that the degree of tumour cell differentiation correlated with over-expression of *IGFBP-2* in hepatoblastoma (Akmal et al, 1995). In their study, expression of *IGFBP-2* was high in poorly differentiated hepatoblastoma and low in well differentiated hepatoblastoma. We examined the levels of expression for both *IGFBP-1* and *IGFBP-2*. The results of this analysis are shown in Figure 4.

Expression of *IGFBP-1* was shown to be decreased in most tumours with the exceptions being samples HB3 and HB6. The overall trend, however, appears to show greatly decreased expression of *IGFBP-1* in hepatoblastomas (Figure 4A). Owing to the limited amounts of tissue available we were unable to include fetal liver in this analysis. *IGFBP-2* expression was also decreased in hepatoblastomas although the degree of the decrease varied. Some samples (HB1, HBs 5–8, HB10 and HB11) showed large decreases in *IGFBP-2* expression, whereas others (HBs 2–4, and HB9) showed moderate or almost normal expression of *IGFBP-2* (Figure 4B). The samples with low expression of *IGFBP-2* have similar expression levels as fetal liver (Figure 4B), whereas those with moderate expression are clearly reduced from that of matched normal liver (Figure 4B).

### DISCUSSION

The IGF-axis plays an important role in many diverse cellular functions including promotion of cell growth and cell survival. Two genes encoding for insulin-like growth factors have been identified, *IGF1* and *IGF2*. The main producer of circulating IGF-I and IGF-II is the liver, and the ability of these peptides to mediate mitogenic, anti-apoptotic and differentiation signals is likely to be primarily via the IGF-IR (Rosen and Pollak, 1999). Regulation of IGF-action is controlled in part by a family of proteins called the insulin-like growth factor binding proteins. This family consists of six high affinity IGFBPs and nine low affinity IGFBP-rPs (Wetterau et al, 1999), each of which shows different tissue specific production and regulatory functions (Rechler and Clemmons, 1998). One of the major functions of IGFBPs is to bind IGFs. By doing so, they form biologically inactive complexes which modulate IGFs from binding to their receptors. The expression of two members of the IGF-axis have previously been shown to be altered in hepatoblastomas (Akmal et al, 1995; Li et al, 1995). If such changes are important in the tumorigenesis or pathogenesis of this disease, a more detailed examination of the IGF-axis in hepatoblastoma may provide greater insights into this disease. In this study we have examined a number of genes from the IGF-axis, including the *IGF1* and *IGF2*, their receptors (*IGF1R* and *M6P*/*IGF2R*), and two members of the IGFBPs (*IGFBP-1* and *IGFBP-2*) in a series of hepatoblastomas. The results were compared to the expression levels for fetal liver and if

*© 2000 Cancer Research Campaign*

**Table 3** Results of the gene expression analysis in the hepatoblastomas with respect to the average fetal liver expression

| Case | Histology | *IGF2* | *H19* | *IGF1* | *IGF1R* | *IGFBP-2* |
|---|---|---|---|---|---|---|
| HB1 | Epithelial | ↓ | ↓ | ↓ | ↓ | N |
| HB2 | Epithelial | ↓ | ↓ | ↓ | ↓ | ↑ |
| HB3 | Epithelial | N | ↑ | ↑ | ↑ | ↑ |
| HB4 | Mixed epithelial/mesenchymal | ↑ | ↓ | ↓ | ↓ | ↑ |
| HB5 | Epithelial | ↓ | ↓ | ↑ | ↑ | ↓ |
| HB6 | Fetal | ↓ | N | ↓ | ↓ | ↑ |
| HB7 | Fetal | ↓ | ↓ | ↑ | ↑ | N |
| HB8 | N/A | ↓ | ↓ | N | N | ↑ |
| HB9 | Fetal | ↓ | ↓ | N | N/D | ↑ |
| HB10 | Epithelial | ↓ | N | ↓ | N | ↑ |
| HB11 | Mixed epithelial/mesenchymal | ↓ | ↓ | ↑ | N | N |

Symbols used: N/D – not determined; N – normal; ↑ – increased expression; ↓ – decreased expression.

available to matched normal liver obtained from the affected individuals at surgery. In this way we could see if expression differences at the individual level were related to the malignancy, and as hepatoblastomas often share similarities to fetal hepatocytes the results could also be compared to fetal liver. Our results demonstrate that in the hepatoblastomas, the expression of many of the IGF-axis genes are altered. An overview of these results is given in Table 2.

Six of the hepatoblastoma samples had increased expression of *IGF1*. Of these six samples, five also had increased *IGF1R* mRNA levels. Thus the increased levels in these samples may be functioning to promote tumour growth and suppress apoptosis. Of the samples showing increased *IGF1* and *IGF1R* mRNA, three of these (HBs 3, 5 and 7) have also been shown to have specifically increased mRNA levels of important cell cycle regulators, growth factors and cyclin-dependent kinase inhibitors (Gray et al, manuscript submitted). In addition, two of these samples have an up-regulation of three genes whose products have been shown to be involved in apoptosis (Gray et al, manuscript submitted). Thus, there may be a competition between apoptotic signals (increased p21, TGF-β and IGFBP-3) and anti-apoptotic signals (increased IGF-I) in these tumours.

The expression of *IGF2* was also observed to be altered in the hepatoblastomas. Of the 11 tumours available nine of these showed an increased expression of *IGF2*. Only one sample (HB6) showed a decrease in the levels of mRNA for this gene. This case is unusual as it shows reduced expression of all genes except one *IGFBP-1* (Table 2).

When the levels of expression of the *M6P*/*IGF2R* receptor were examined, most of the tumours showed decreased or normal levels of mRNA for this gene. As one of the functions of the product of this gene is to bind IGF-II for subsequent internalization and degradation by lysosomes (De Souza et al, 1997), an increase in expression of *IGF2* without a concommitant increase in the expression of *M6P*/*IGF2R* may indicate that the cells in these tumours have an increased mitogenic potential due to the increase in *IGF2* and *IGF1*. In some situations this mitogenic potential is further increased by having increased levels of *IGF1R* (increased signalling potential) most notably HB3 and HB5. When levels of *IGFBP-1* and *IGFBP-2* mRNA were examined, the results show that for nearly all samples, expression of these genes are reduced in the tumours. IGFBP-1 has been shown to be the predominant IGFBP in amniotic fluid and fetal plasma. In the liver, expression of this gene in parenchymal cells has been demonstrated. Low levels of IGFBP-1 protein have been correlated with fetal overgrowth (Spagnoli and Rosenfeld, 1997). Nine of 11 tumour samples show decreased expression of *IGFBP-1* mRNA. Therefore the reduction in the mRNA may be reflected in the protein levels, leading to increased tumour growth potential, in a manner similar to that observed for fetal overgrowth. However, one of the hepatoblastomas classified as a fetal type, shows normal *IGFBP-1* mRNA levels when compared to its matched normal liver. As no examination of *IGFBP-1* mRNA in fetal tissue was carried out in this study, a distinct correlation between the levels of this genes mRNA to overgrowth cannot be assumed. Further experiments should be carried out including immunohistochemistry for this protein to see if such a correlation exists. IGFBP-2 has been shown to be produced in the liver by Kupffer and parenchymal cells. An early report by Ikeda and collegues showed that *IGFBP-2* expression was altered in hepatoblastomas. This study showed that well differentiated tumours and normal liver had no detectable *IGFBP-2* or *IGF2* expression, whereas poorly differentiated tumours had high expression of *IGFBP-2* (Akmal et al, 1995). In contrast to this report we have shown that normal liver produces comparably large quantities of detectable *IGFBP-2* mRNA (Figure 4B). One of the functions of the IGFBPs is to bind IGFs, and by doing so they form biologically inactive complexes which affect the the ability of the IGFs to bind to its receptors. As *IGFBP-1* and *IGFBP-2* mRNA is reduced in most tumours, excess biologically active IGFs may therefore be available in these tumours to potentiate proliferative effects. In addition to these IGFBPs, we have also examined the expression of *ALS*, two other *IGFBPs* (*IGFBP-3* and *IGFBP-5*) and *IGFBP-rP1* in these tumours (Gray et al, manuscript submitted, data not shown). The results show that the levels of all of these genes are affected in these tumours.

In addition to examining the IGF-axis we also examined the expression of the potential tumour suppressor *H19*, in these samples. In ten of the 11 samples, expression of this gene was reduced. As one of the proposed functions of this gene is to suppress growth, down-regulation of the gene may therefore predispose the hepatoblastomas to overgrowth. This may be

 *© 2000 Cancer Research Campaign*

particularly important for the tumour HB1. In this tumour expression of every gene except *IGF2* is decreased (Table 2). In this sample expression of *IGF2* is normal, but levels of *M6P/IGF2R*, *IGFBP-1* and *IGFBP-2* are decreased, and *H19* is also greatly decreased. Thus, there may be increased levels of active IGF-II to potentiate growth signals, and the lack of *H19* expression may amplify this response.

The alterations in expression of members of the IGF-axis do not appear to correlate with the clinical outcome of this disease. Only one patient failed to achieve clinical remission, and the expression profile for this individual (HB2), is similar to that for another individual (HB4), who shows no evidence of the disease following surgical intervention (Table 2). There also appears to be no correlation between gene expression differences and tumour type as no tumour type can be separated from the others on the basis of gene expression differences examined here (Table 2). It may be argued that the expression patterns observed indicate a tendency towards a fetal liver phenotype.

When the expression profiles against the average fetal liver expression were compared, we were unable to discover any such correlations (Table 3). One might also argue that there may be normal cells present within some of the tumour samples which may affect the analysis by masking any alterations in expression. This may be especially true for those samples in which gene expression from the tumour is similar to that observed for normal liver (Table 2). In such cases, a more comprehensive analysis could be determined using techniques such as in situ hybridization. Alternatively micro-dissection of the tumours may provide tumour rich mRNA for analysis. Such studies are in the process of being initiated.

In conclusion, the IGF-axis is affected in hepatoblastomas. While there are no definitive explanations on the role these alterations may play in the tumorigenesis process, one potential result of these alterations may be that local concentrations of IGFs in combination with reduced levels of IGFBPs promote clonal expansion of the tumour cells. Further studies are indicated for in order to determine the exact importance of the IGF-axis in hepatoblastomas.

### REFERENCES

Akmal SN, Yun K, MacLay J, Higami Y and Ikeda T (1995) Insulin-like growth factor 2 and insulin-like growth factor binding protein 2 expression in hepatoblastoma. *Hum Pathol* **26**: 846–851

Albrecht S, von Schweinitz D, Waha A, Kraus JA, von Deimling A and Pietsch T (1994) Loss of maternal alleles on chromosome arm 11p in hepatoblastoma. *Cancer Res* **54**: 5041–5044

Chomczynski P and Sacchi N (1987) Single-step method of RNA isolation by acid guanidinium thiocyanate-phenol-chloroform extraction. *Anal Biochem* **162**: 156–159

Constancia M, Pickard B, Kelsey G and Reik W (1998) Imprinting mechanisms. *Genome Res* **8**: 881–900

De Souza AT, Yamada T, Mills JJ and Jirtle RL (1997) Imprinted genes in liver carcinogenesis. *FASEB* **11**: 60–67

Ekström TJ, Cui H, Li X and Ohlsson R (1995) Promoter-specific IGF2 imprinting status and its plasticity during human liver development. *Development* **121**: 309–316

Franklin GC, Adam GI and Ohlsson R (1996) Genomic imprinting and mammalian development. *Placenta* **17**: 3–14

Li X, Adam G, Cui H, Sandstedt B, Ohlsson R and Ekström TJ (1995) Expression, promoter usage and parental imprinting status of insulin-like growth factor II (IGF2) in human hepatoblastoma: uncoupling of IGF2 and H19 imprinting. *Oncogene* **11**: 221–229

Li X, Gray SG, Flam F, Pietsch T and Ekström TJ (1998*a*) Developmental-dependent DNA methylation of the IGF2 and H19 promoters is correlated to the promoter activities in human liver development. *Int J Dev Biol* **42**: 687–693

Li X, Kogner P, Sandstedt B, Haas OA and Ekström TJ (1998*b*) Promoter-specific methylation and expression alterations of igf2 and h19 are involved in human hepatoblastoma. *Int J Cancer* **75**: 176–180

Looijenga LH, Verkerk AJ, De Groot N, Hochberg AA and Oosterhuis JW (1997) H19 in normal development and neoplasia. *Mol Reprod Dev* **46**: 419–439

Montagna M, Menin C, Chieco-Bianchi L and E D'Andrea (1994) Occasional loss of constitutive heterozygosity at 11p15.5 and imprinting relaxation of the IGFII maternal allele in hepatoblastoma. *J Cancer Res Clin Oncol* **120**: 732–736

Morison IM and Reeve AE (1998) Insulin-like growth factor 2 and overgrowth: molecular biology and clinical implications. [Review]. *Mol Med Today* **4**: 110–115

Odell SD and Day IN (1998) Insulin-like growth factor II (IGF-II). *Int J Biochem Cell Biol* **30**: 767–771

Ohlsson R, Hedborg F, Holmgren L, Walsh C and Ekström TJ (1994) Overlapping patterns of IGF2 and H19 expression during human development: biallelic IGF2 expression correlates with a lack of H19 expression. *Development* **120**: 361–368

Olivecrona H, Hilding A, Ekström C, Barle H, Nyberg B, Moller C, Delhanty PJ, Baxter RC, Angelin B, Ekström TJ and Tally M (1999) Acute and short-term effects of growth hormone on insulin-like growth factors and their binding proteins: serum levels and hepatic messenger ribonucleic acid responses in humans. *J Clin Endocrinol Metab* **84**: 553–560

Rainier S, Dobry CJ and Feinberg AP (1995) Loss of imprinting in hepatoblastoma. *Cancer Res* **55**: 1836–1838

Rechler MM and Clemmons DR (1998) Regulatory actions of insulin-like growth factor binding proteins. *Trends Endocrinol Metab* **9**: 176–183

Rosen CJ and Pollak M (1999) Circulating IGF-I: new perspectives for a new century. *Trends Endocrinol Metab* **10**: 136–141

Sainati L, Leszl A, Stella M, Montaldi A, Perilongo G, Rugge M, Bolcato S, Iolascon A and Basso G (1998) Cytogenetic analysis of hepatoblastoma: hypothesis of cytogenetic evolution in such tumors and results of a multicentric study. *Cancer Genet Cytogenet* **104**: 39–44

Smrzka OW, Fae I, Stoger R, Kurzbauer R, Fischer GF, Henn T, Weith A and Barlow DP (1995) Conservation of a maternal-specific methylation signal at the human IGF2R locus. *Hum Mol Genet* **4**: 1945–1952

Spagnoli A and Rosenfeld RG (1997) Insulin-like growth factor binding proteins. *Curr Opin Endocrinol Diabetes* **4**: 1–9

Wetterau LA, Moore MG, Kuk-Wah L, Shim ML and Cohen P (1999) Novel aspects of the insulin-like growth factor binding proteins. *Mol Genet Metab* **68**: 161–181

von Schweinitz D, Wischmeyer P, Leuschner I, Schmidt D, Wittekind C, Harms D and Mildenberger H (1994) Clinico-pathological criteria with prognostic relevance in hepatoblastoma. *Eur J Cancer* **30A**: 1052–1058

Yun K, Jinno Y, Sohda T, Niikawa N and Ikeda T (1998) Promoter-specific insulin-like growth factor 2 gene imprinting in human foetal liver and hepatoblastoma. *J Pathol* **185**: 91–98

*© 2000 Cancer Research Campaign* 
